# Changes in land use enhance the sensitivity of tropical ecosystems to fire-climate extremes

**DOI:** 10.1038/s41598-022-05130-0

**Published:** 2022-01-19

**Authors:** Sujay Kumar, Augusto Getirana, Renata Libonati, Christopher Hain, Sarith Mahanama, Niels Andela

**Affiliations:** 1 Hydrological Sciences Laboratory, NASA Goddard Space Flight Center, Greenbelt, MD USA; 2 Science Applications International Corporation, Greenbelt, MD USA; 3 Instituto de Geociência, Universidade Federal do Rio de Janeiro, Rio de Janeiro, RJ Brazil; 4 Short-Term Prediction Research and Transition Center, NASA Marshall Space Flight Center, Huntsville, AL USA; 5 School of Earth and Environmental Sciences, Cardiff University, Cardiff, Wales

**Keywords:** Hydrology, Environmental impact, Natural hazards, Environmental health

## Abstract

The Pantanal, the largest contiguous wetland in the world with a high diversity of ecosystems and habitat for several endangered species, was impacted by record-breaking wildfires in 2020. In this study, we integrate satellite and modeling data that enable exploration of natural and human contributing factors to the unprecedented 2020 fires. We demonstrate that the fires were fueled by an exceptional multi-year drought, but dry conditions solely could not explain the spatial patterns of burning. Our analysis reveals how human-caused fires exacerbated drought effects on natural ecosystem within the Pantanal, with large burned fractions primarily over natural (52%), and low cattle density areas (44%) in 2020. The post-fire ecosystem and hydrology changes also had strong ecological effects, with vegetation productivity less than − 1.5 σ over more than 30% of the natural and conservation areas. In contrast to more managed areas, there was a clear decrease in evaporation (by ~ 9%) and an increase in runoff (by ~ 5%) over the natural areas, with long-term impacts on ecosystem recovery and fire risk. This study provides the first tropical evidence outside rainforests of the synergy between climate, land management and fires, and the associated impacts on the ecosystem and hydrology over the largest contiguous wetlands in the world.

## Introduction

Vegetation disturbance from fires is an essential component of savanna and woodland ecosystems^1^. Fires clear older biomass, release nutrients, and open the canopy, stimulating new vegetation growth^2–4^. Irrespective of the ignition source, fires are often exacerbated by climate conditions such as warmer and drier conditions, drought, and heatwaves which increase vegetation flammability. In the contemporary tropics, most ignitions are from human origin^5^. Human influence in triggering and redistributing ignitions are well established over different areas^6,7^, with 84 to 97% of the wildfires in the United States being humans driven^8,9^, only 13% of the bushfires in Australia from natural causes^6^, and similar trends in other places in the world^10^. In contrast to global savanna^11^, the increase in managed land from deforestation, forest clearing for livestock grazing, and climate-change driven changes in extremes combined with increases in anthropogenic ignitions pose an increasing threat to the sustainability of tropical forests and woodlands^12–17^. Commensurate with the increase in global temperatures, droughts have been more severe^18–21^, with approximately 19% increase in the mean fire weather season length globally attributed to climate change variations^22^. These novel climate conditions have resulted in the emergence of a new fire type, synchronized fire events that affect significant portions of entire landscapes^23,24^ in different parts of the world. The magnitude of these recent events has fundamental consequences for trajectories of post-fire recovery, through alterations of hydrological fluxes and regional climate.

Coincident with the extreme droughts^25–29^, biomes in Brazil have been hotspots for forest fires over recent decades^22,30^. Similar to the fire occurrences in other parts of the world, human initiation is attributed to most fires in the humid tropical forest ecosystems of the Amazon^16,31^. The industrial agriculture and cattle ranching induced forest clearing with fires^32,33^ have increased the frequency of understory fires in recent years^34^ and fire might become the dominant driver of forest degradation under scenarios of future change^35^.

**Figure 1:**
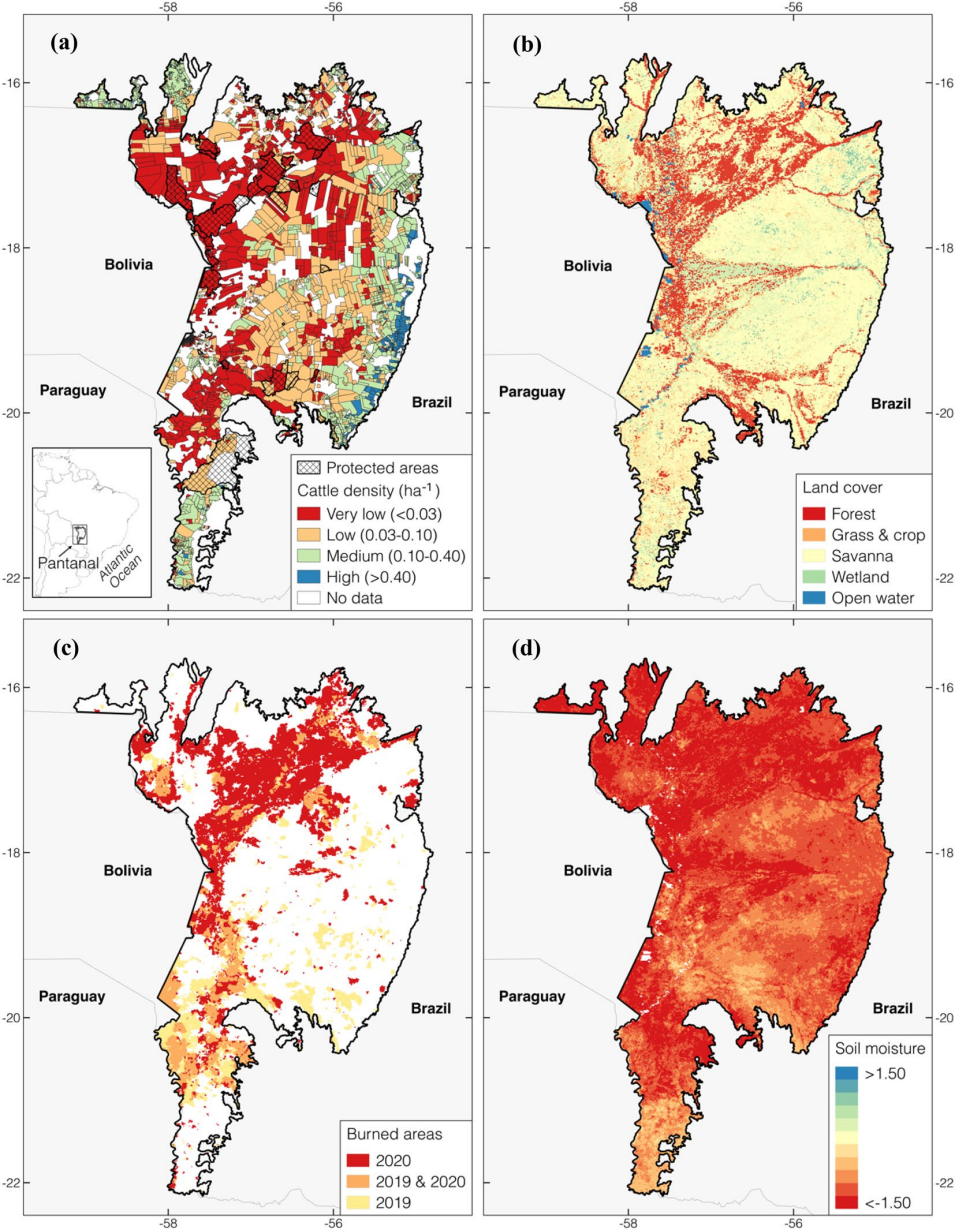
Map of the study domain over the Pantanal: (**a**) shows four categories of pasture based on cattle head density per cattle ranch (estimated from the Mapbiomas project^37^), with the conservation areas (shown in the hatched patterns) with the indigenous and environmental reserves (http://www.funai.gov.br/index.php/shape; https://antigo.mma.gov.br/areas-protegidas/cadastro-nacional-de-ucs/dados-georreferenciados.html), (**b**) shows the four dominant land cover types from the MODIS land cover data^67^, (**c**) shows the locations of the burned areas in 2019 and 2020, based on the MCD64A1 burned area product^43^, and (**d**) shows a map of the standardized 6-month root zone soil moisture anomaly averaged across the Aug-Nov of 2020. The spatial maps are created using QGIS (https://qgis.org/en/site/).

The Pantanal is a region in central South America consisting of the largest contiguous wetland in the world (Fig. 1), located mostly in the Brazilian state of Mato Grosso do Sul. The Pantanal hosts the highest concentration of wildlife in South America, which is sustained by the floodplains that are submerged in the rainy season and drained in the dry season^36^. Cattle ranching is a major part of the Pantanal economy, and 93% of the land on the Pantanal is private, which is where the industrialized farming typically occurs. The region also includes protected areas and indigenous lands, over which cattle ranching is minimal^37^. Despite the long history of the use of fires to clear the area for grazing, large-scale vegetation density in the region has largely remained unaffected by such activities^38^, particularly over the conservation areas. The Pantanal has been hit by an unprecedented drought since 2018^39,40^, followed by extreme wildfires^41,42^. Here, we used burned area estimates from the Moderate Resolution Imaging Spectroradiometer (MODIS) sensor aboard the NASA Terra and Aqua satellites (MCD64A1^43^), and found that approximately 3.7 Mha burned, representing 28.9% of the region burned from the 2020 fires (Fig. 1c), corroborating data from the ALARMES warning system^44^ from the Laboratory for Environmental Satellite Applications (LASA-UFRJ; https://lasa.ufrj.br/alarmes). The exceptional nature of these fires has raised widespread concerns about the conservation of the Pantanal landscape^42,45,46^, but much remains unknown about the precise drivers of the event and its long-term implications.

In this study, we use state-of-the-art land surface modeling and remote sensing data assimilation techniques to understand processes that led to the 2020 Pantanal fires and their impacts. First, using a land surface modeling integration^47^ that incorporates information from remote sensing inputs of vegetation changes, we assess the severity, duration, and extent of the recent drought. Second, we use remotely sensed estimates of burned areas to examine the changing influence of drought on fire instances over time and how the historical occurrences of fires increase over the natural areas and reduce over heavily managed regions. We combine model results with data about cattle density and land cover (Fig. 1a-b) to identify the outlier nature of the 2020 fires and interpret its drivers. Finally, we use the land surface model integration to assess the nature of the changes in the ecosystem and regional hydrology as a result of the fires by quantifying the unprecedented nature of the changes in vegetation, ecosystem productivity, and hydrology due to the fire-induced removal of vegetation. Combined, these analyses highlight how human activity in recent years has disproportionately made natural systems of the Pantanal more prone to fire occurrences during extreme droughts.

### The driving factors behind the 2020 Pantanal fires

Over 71% of the region, about 9.2 Mha, face standardized root zone soil moisture anomaly values below − 1 $\sigma$, indicating the unprecedented nature of the 2020 drought, both in terms of the magnitude and spatial extent (Fig. 1d). We examine the severity and extent of the recent drought over the Pantanal using deficits in antecedent precipitation and root zone soil moisture as analogs of drought conditions. Precipitation shortages represent the meteorological factors driving the anomalous dry conditions. Deficits of soil moisture resulting from the imbalance between moisture supply on the land surface and the losses from evaporation and runoff are typically used to characterize agricultural droughts^48–50^. Maps of root zone soil moisture anomalies for the dry season (Aug-Nov) across 2017 to 2020 (Fig. S1) indicate that, prior to 2018, the small root zone soil moisture anomalies are representative of the typical dry season. The drought onset is noticed in 2018, and by 2019 much of the region is in consistent negative soil moisture anomalies, exacerbating in 2020.

**Figure 2:**
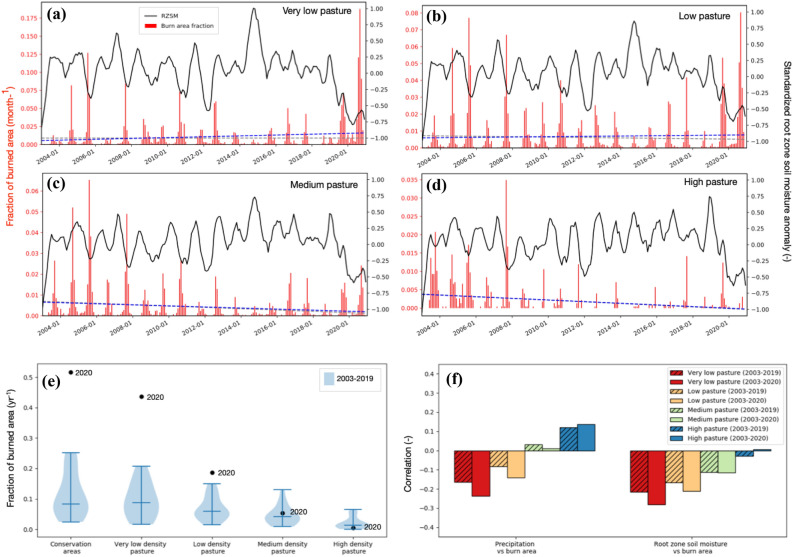
Time series of the standardized anomalies of antecedent 6-month root zone soil moisture (black lines) and fraction of burned areas per month (red bars) over areas with different pasture density (panels **a**, **b**, **c**, **d**). The dashed blue and gray lines show the linear trend of the monthly fraction of burned area (Table 1) over the 2003–2020 and 2003–2019 time periods, respectively. Panel e shows the distribution of annual fraction of burned area across 2003–2019 and from 2020 and Panel f shows the Spearman correlation between the standardized anomalies in variables relevant for fuel moisture (i.e., 12-month precipitation, 6-month root zone soil moisture) and percent of burn area, stratified for areas with four different levels of pasture density.

Historical trends (2003–2020) in MODIS-burned area^43^ stratified by cattle density (Fig. 2) and land cover type (Fig. S2) highlight the influence of the land use changes on the 2020 fires. Strikingly, we found that under similar drought conditions, natural landscapes were most sensitive to fire extremes. During 2020, 52% (362% increase compared to long-term mean) of protected areas and 44% (357% increase) of areas with very low cattle density burned, compared to 6% (16% increase) and 0.5% (8% decrease) of areas with medium and high cattle density, respectively (Fig. 2e). Similarly, 52% of all forests (692% increase compared to the long-term mean), 31% (814% increase) of wetlands have burned in 2020, compared to 27% (200% increase) of the grass and croplands and 21% (125% increase) of savannas, areas that are, to a large part, used for grazing (Fig. S2e). These patterns are also reflected in the historical time series, while there is a general correspondence between negative root zone soil moisture anomalies and the fire occurrences over the very low-density pasture areas, the strength of this relationship decreases with increasing cattle density (Fig. 2f).

The sensitivity of protected and extensively used land to the 2020 extreme fire occurrence is further highlighted by time series analysis. Figure 2f shows that the correlation between antecedent precipitation and burned area range from − 0.16 to 0.12 and the root zone soil moisture and burned area range from − 0.21 to − 0.03 during 2003–2019. Over the very low pasture areas, the association between precipitation and burned areas increases significantly (from − 0.16 to − 0.24) when 2020 data are considered, whereas no considerable change in the medium and high pasture density areas is seen. These results highlight the extreme and unequal impact of the 2020 drought, but also suggest that the sensitivity of natural ecosystems to fire has increased over time. Indeed, the observed trends in fraction of burned areas in the very low, low, and medium pasture areas were not significant during 2003–2019 (Table 1), but when 2020 data are included, there is a statistically significant increasing trend in the fraction of burned area over the protected and very low-density pasture areas. In contrast, no change is observed over the low and medium density areas where the relationship between burned area and drought remains weak. It is also notable that a statistically significant decreasing trend in the fire occurrences is seen over the high-density pasture areas, which is unaffected by the inclusion or exclusion of the 2020 data, consistent with increasing industrialization and cattle densities over time.

**Table 1:** Slope of the trendlines of monthly fraction of burned area over areas (month^−1^) with different levels of pasture density and land cover type.

	2003–2019	Trend	2003–2020	Trend
Protected areas (1.31 Mha)	2.2E−5	No trend	1.4E−3	Increasing
Very low pasture density (4.22 Mha)	− 1.2E−6	No trend	4.92E−3	Increasing
Low pasture density (3.59 Mha)	− 9.93E−4	No trend	8.22E−4	No trend
Medium pasture density (1.95 Mha)	− 2.67E−3	No trend	− 2.13E−3	No trend
High pasture density (0.22 Mha)	− 1.95E−3	Decreasing	− 1.85E−3	Decreasing
Forests (2.57 Mha)	− 1.73E−4	No trend	6.50E−3	Increasing
Wetlands (0.60 Mha)	1.16E−3	No trend	5.24E−3	Increasing
Grass and Crops (2.63 Mha)	− 9.25E−4	No trend	1.98E−3	No trend
Savannas (6.99 Mha)	− 8.81E−4	No trend	9.16E−4	No trend

The statistical significance of the trends is also indicated in the table.

Similar results are obtained with comparisons of the fraction of burned areas and the associated root zone soil moisture anomalies over the four main land cover types (Fig. S2). The forests and wetlands are mostly natural areas whereas the crop, grass, and savanna areas are, to a large part, used for grazing. The natural vegetation areas show significantly higher percent of burns in 2020 compared to such occurrences in the observational record. On the other hand, the fraction of the burned areas over the crop, grass and savanna areas, is similar to the historical period prior to 2020. The anomalous nature of the fires is also seen over the wetlands, where fire occurrences are rare and range around 2%. In 2020, however, more than 10% of the wetlands burned. Our analysis quantifies that as much as 45% of the conserved areas have burn scar features in late 2020, significantly larger than the fire occurrences in the MCD64A1 data record. In the years prior to 2020, the percent of burns in the conserved areas never exceeded 18%.

Patterns similar to Figure 2f are observed when the level of association between antecedent moisture conditions and the fraction of burned area is stratified by the main land cover types and over the conservation and non-conservation areas (Fig. S3). Compared to the 2003–2019 period, there is a statistically significant change in the correlation between antecedent precipitation with the percent of burned area over the forests (a decrease of 36%) and wetlands (a decrease of 52%) when data from year 2020 is included (Fig. S3a). On the other hand, over the crop, grass and savanna areas, the changes in correlation between the antecedent precipitation anomalies and burned area with and without 2020 are not statistically significant. Similar patterns are observed between areas classified as reserves and non-reserves (Fig. S3b). Over the reserves, there is a statistically significant increase in the association between antecedent precipitation and the fraction of burned areas, when the 2020 record is considered, whereas over non-reserves no statistically significant change in the fire activity is observed for 2020.

We also examined the influence of large climate patterns (Fig. S3c) by examining the changes in correlation between the percentage of burned areas with the climate indices of the Atlantic Multidecadal Oscillation (AMO^51^), the Pacific Decadal Oscillation (PDO^52^) and the Multivariate El Nino Index (MEI^53^). The AMO represents the anomalous warming of the Atlantic Ocean, whereas PDO and MEI represent the anomalous warming of the equatorial and eastern tropical Pacific and tropical north Atlantic oceans, respectively. Consistent with prior studies over Amazon^31,54^ and Pantanal^40^, there is a general association between these climate indices and the drought and fire instances in the Pantanal. Though these correlation values show a marginal change when the year 2020 is included, compared to the period of 2002–2019, these changes are not statistically significant, suggesting that the increase in fire activity in 2020 is not solely explained by the anomalies in large scale climate variability indices. In contrast to the widely held assumption that remote areas are well protected from ecosystem degradation, our results highlight how the natural environments are more prone to fires under extreme drought conditions and human-induced ignitions, such as in 2020. Under such scenarios, fires spread over large areas without any suppression, increasing the difficulty of controlling fires^42^. Conservation strategies such as domesticated cattle exclosures will be less effective in such areas, under scenarios of future climate change with more frequent occurrences of dry extremes^55^.

To confirm the inferences from the correlation analysis, a Random Forest (RF) classifier model using the pasture density, landcover type, standardized anomalies of precipitation, root zone soil moisture, Leaf Area Index (LAI), and Gross Primary Production (GPP) as predictors and the burned area locations as the predictand is used (the larger scale climate indicators are not included given their lack of specific influence in the 2020 fires). The ordering of the importance of the predictors is generally consistent (Fig. S4) with the results from the one-variable at a time regression estimates described above, with the pasture density and the antecedent root zone soil moisture anomalies being the predictors with larger importance values. For example, the drop column importance values for the pasture density and antecedent root zone soil moisture are 0.28 each, higher than 0.21 for precipitation, 0.18 for landcover, 0.04 for LAI and 0.0 for GPP. The relative importance of the pasture density from this analysis also points to the role of human management in the fires over the Pantanal. This suggests human-caused fires often escape beyond their intended area in natural landscapes while controlled fires over areas with more grazing and pasture are not necessarily influenced by dry and drought conditions.

### Ecosystem and hydrology impacts from the 2020 Pantanal fires

**Figure 3:**
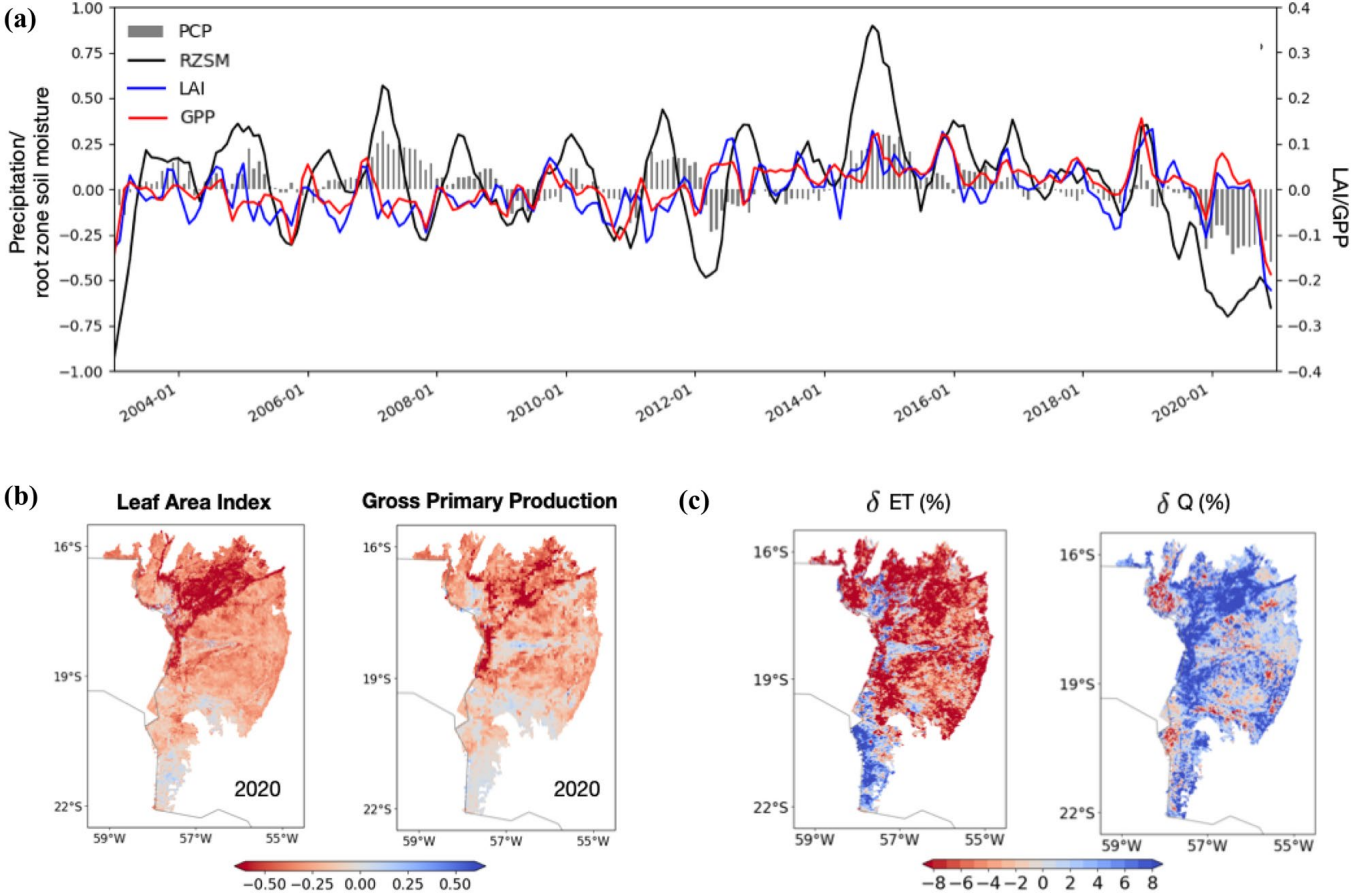
Time series (Panel a) of domain averaged standardized anomalies of 12-month precipitation, 6-month root zone soil moisture (RZSM), 2-month Leaf Area Index (LAI), and 2-month Gross Primary Production (GPP). The bottom panel b shows the ecosystem impacts of the 2020 fire events, with spatial maps of standardized anomalies in 2-month LAI, and 2-month GPP, averaged during Sep-Nov for 2020. Panel c shows impacts of fires on the local hydrology over the Pantanal. The percentage change in evapotranspiration (ET) and runoff (Q) during the post-fire time period, relative to a scenario where climatological average vegetation conditions are present. The spatial maps in Panels b and c are created using matplotlib (https://matplotlib.org/).

The impact of fires on the ecosystem and hydrological conditions had strong ecological effects, though the exceptional drought is largely uniform across the whole region. Except for 2020, the correspondence between drought and negative vegetation anomalies is weak in this region (Fig. 3a; S5a-b), indicating that the Pantanal ecosystem is very resilient to moisture stress conditions. Moreover, while the entire region is in drought, the significant drop in LAI and GPP is primarily observed over areas such as the forests, wetlands, and conservation areas (Fig. 3b), with close correspondence to burned areas during 2020. Over the non-forested areas, a more moderate drop in LAI and GPP is observed. The results of this study also show that changes in precipitation are not the main driver of vegetation variability, consistent with prior studies that indicated that rainfall is not a good predictor of local vegetation structure in areas that flood seasonally^56^. The correlation between the standardized LAI and GPP anomalies and antecedent precipitation anomalies (Fig. S5a-b) is generally small (< 0.10), particularly in the 2002–2019 period. The significant negative anomalies in precipitation and vegetation states in 2020 increase these associations when the entire period is considered. This pattern is observed in the stratification with the dominant vegetation types and the pasture areas.

Our results show that antecedent vegetation conditions are not a major factor in the development of fires, rather, negative vegetation anomalies are observed following the fires (Fig. S5c-d). Compared to precipitation and root zone soil moisture, the antecedent vegetation anomalies are more weakly correlated with the burn areas. There is no statistically significant relationship between the antecedent LAI and burned areas prior to 2020, whereas the correlation between antecedent GPP and burned areas show little change with and without 2020, both over different land cover and pasture regimes. This is an indication that the potential fuel loads from the negative vegetation anomalies are not a major factor in the fire occurrences. On the other hand, the 2-month leading LAI and GPP anomalies show stronger correlations with the burned areas, indicating that the negative anomalies in vegetation variables follow the fire related burns or are consequences of the fires. The changes in the correlation between leading LAI/GPP and burn area in the 2002–2019 and 2002–2020 are statistically significant over the forests, confirming that the vegetation disturbances follow the fires over these areas. Over the crop, grassland, and savannas, the change in the correlations with and without 2020 is not statistically significant. During the 2003–2019 period, the average % of conservation areas with standardized LAI (GPP) values below − 1.5 $\sigma$ is only 3.3 (1.5), whereas in 2020, the % of areas with such large vegetation alterations jump to 35.6 (28.2) (Table 2). Similar large changes in vegetation anomalies are seen in areas with historically very low pasture density and forests, whereas over other areas, more moderate reductions in vegetation productivity are seen.

**Table 2:** Standardized anomalies of LAI and GPP and the percentage of area where the standardized anomalies are less than − 1.5 σ, stratified over conservation areas and regions with different pasture density and land cover types, averaged across 2003–2019 and for 2020.

	LAI (standardized anomaly, % of area below – 1.5 σ)		GPP(standardized anomaly, % of area below – 1.5 σ)	
	2003–2019	2020	2013–2019	2020
Conservation areas	0.02,3.3	− 0.23,35.6	0.02,1.5	− 0.10,28.2
Very low pasture density	0.00,3.5	− 0.24,30.8	0.02,2.3	− 0.10,26.8
Low pasture density	− 0.02,2.9	− 0.11,14.4	0.03,2.4	− 0.03,18.1
Medium pasture density	− 0.02,1.9	− 0.14,6.3	0.02,1.6	− 0.02,10.1
High pasture density	− 0.03,1.3	− 0.11,2.0	0.01,0.1	0.01,4.1
Forests	0.02,5.7	− 0.47,55.3	0.03,1.9	− 0.19,41.4
Wetlands	0.06,3.4	− 0.03,25.8	0.00,1.6	− 0.03,26.5
Grass and Crops	− 0.02,3.2	− 0.10,17.0	0.02,2.5	− 0.04,20.3
Savannas	− 0.02,2.1	− 0.10,10.4	0.02,2.3	− 0.03,14.8

The annual average precipitation in 2020 was 40% below average fueling the drought and wildfires. A number of precipitation events in late October and early November, 2020 eventually helped in extinguishing the fires. During those rain events, the changes in ET and runoff had strong ecological effects with increasing runoff (with the interquartile range of 3–9%) and reduced ET (with the interquartile range of 0 to − 15%) over the burned areas of forests, conservation, and very low-density pasture (Fig. 3c), confirming that the removal of vegetation due to fire impacted the local hydrological response. Over other areas, the magnitude of such changes is smaller with a net impact of no clear shift in the hydrological regime (Fig. S6). Note that the increasing runoff and reducing ET impact from fires quantified here is likely underestimated, as factors such as water repellence of the soil from fires^57^, are not represented in our modeling system. As a consequence of the increased runoff, increased erosion and sediment yield can further contribute to the land degradation. The drastic changes in the local hydrology from the 2020 fires in the Pantanal underscore how the combination of droughts and human caused fires can have long lasting effects on the hydrological cycle and regional climate. These changes in turn form part of a positive feedback loop, with drier and warmer conditions, more open cover types, and enhanced fire risk.

### Implications

Understanding the complex feedback mechanisms involving fire, climate, vegetation, and human activity is crucial, but can only be achieved when there is comprehensive knowledge about these patterns. The sensitivity of natural landscapes to fire-driven degradation has been a concern across the southern Amazon for years, but here we demonstrate that the same mechanisms may be more universally applicable across the tropics. This study provides the first tropical evidence outside rainforests of the synergy between climate, land management and fires, and the associated impacts on the ecosystem and hydrology over the largest contiguous wetlands in the world. Though similar levels of drought anomalies have been observed in the past decades, the temporal extent of the 2020 drought exceeds what has been observed in prior years. Nevertheless, climate alone could not explain the unprecedented extent and location of wildfires in the Pantanal, in particularly during 2020, as previous observed over the Amazon rainforest^16,31^. Here, we demonstrate how cattle density, and associated shifts in land use drive regional fire patterns and its sensitivity to climate. Strikingly, natural areas, not human-dominated landscapes, were most sensitive to fire driven ecosystem degradation. While such conditions have been predicted to occur across parts of the southern Amazon^34,35,58–61^, here we provide large-scale evidence from the observational record. In addition, the biome-scale extent of the fires may further slow ecosystem recovery through its impacts on regional hydrology and climate. We observed a 30% decrease in the vegetation productivity, 9% decrease in ET and a 5% increase in runoff across the burned regions presenting an important feedback mechanism^12,62,63^. Our findings have global implications, as they demonstrate how tropical land management and fires impact the regional water cycle. Combined, these two factors can result in rapid degradation of natural areas previously assumed to be protected by their remoteness. Based on these findings, there is an urgent need to rethink tropical ecosystem conservation and adaptation strategies, using a framework that acknowledges the compounding, cascading and long-term effects of land management and climate. The quantification of such long-term impact assessments is left for a future work.

## Methods

### Modeling and data assimilation setup

A combination of physical modeling and machine learning approaches are employed in this study to examine the anomalous nature of the exceptional 2020 drought over the Pantanal and to identify their contribution to exacerbating the fires in this region. The Noah-MP land surface model (version 4.0.1^47,64^) is forced with the surface meteorology from NASA’s Modern Era Retrospective-Analysis for Research and Applications, version 2 (MERRA2^65^) and precipitation data from NASA’s Global Precipitation Measurement (GPM) Integrated Multi-satellitE Retrievals for GPM (IMERG^66^). MERRA2 data is available at hourly intervals and approximately about 50-km spatial resolution whereas IMERG provides precipitation data at every 30-minute intervals at, approximately, 10-km spatial resolution. The land surface model (LSM) simulations are conducted at 1-km spatial resolution over the domain shown in Fig. 1. The model configuration employs land cover data (at 1-km resolution) from the modified International Geosphere Biosphere Program (IGBP) Moderate Resolution Imaging Spectroradiometer (MODIS^67^) and soil parameters (at 1-km resolution) from the International Soil Reference and Information Centre (ISRIC^68^), and topography (at 90m resolution) from the Multi-Error Removed Improved-Terrain (MERIT^69^) digital elevation map. Statistical downscaling approaches are used to transform the coarse resolution MERRA2 and IMERG data to 1-km spatial resolution. The input meteorological fields of air temperature, humidity, surface pressure, wind, downward shortwave radiation, and downward longwave radiation are downscaled to 1-km by adjusting for terrain differences in elevation, slope, and aspect^70^. Precipitation fields at 1-km are generated by using the monthly high resolution WorldClim climatology^71^ to spatially disaggregate IMERG data to 1-km. The initial conditions for the LSM are generated through a long integration of the Noah-MP model starting in 2000. The model is cycled from year 2000 to 2020 and then reinitialized in 2000. All model integrations are conducted using the open source NASA Land Information System (LIS^72^) software.

Among other features, Noah-MP LSM includes a dynamic phenology model^73^ integrated with the Ball-Berry photosynthesis based stomatal resistance model^74^. In this study, we assimilate the 500m MCD15A2H collection 6 leaf area index (LAI) retrievals from the Moderate Resolution Imaging Spectroradiometer (MODIS) sensors aboard NASA’s Terra and Aqua satellites, using this feature in Noah-MP. Similar to the studies that describe the assimilation of MODIS LAI data^75,76^, a 1-dimensional Ensemble Kalman Filter (EnKF) algorithm is used to assimilate the LAI retrievals, with a 20 member model ensemble. The ensemble spread is created by applying small perturbations to the model states and meteorological inputs^75^. During assimilation, the prognostic leaf mass variable in the LSM is updated in response to the LAI inputs from the MODIS data. Before assimilation, the MCD15A2H data is aggregated to 1-km resolution and only data values flagged as ‘good quality’ considering factors such as cloud contamination, algorithm saturation, and detector signal quality are used in assimilation.

To evaluate the impact of the vegetation disturbances from the 2020 Pantanal fires on the local hydrology, an additional integration is conducted where the daily climatological vegetation conditions across the 2002–2019 time period are assimilated into the Noah-MP model (called the LAI-climo DA) during the year 2020. As the difference between the LAI-DA and LAI-climo-DA model runs is solely the vegetation changes from the 2020 fires, these two integrations are used to quantify the influence of the fire driven vegetation changes.

A multi-variable random forest (RF) classifier model^77^ focusing on the local scale variables, was developed to consider the joint or cross influence of the human management, fuel moisture and fuel load variables to fire occurrences over the Pantanal. RF models are non-parametric and are shown to work well with correlated and conditional variables and are robust in the presence of outliers and noise in the data^78–80^. RF also generates measures of feature importance, a score to indicate the usefulness of the input variable at predicting the target. Three different approaches are used to compute the feature importances generated by the RF model. The native approach with RF models computes the feature importances based on how much each feature decreases the variances of the optimality criteria used in the model. Similarly, the permutation feature importance method computes the feature importances by randomly reshuffling while preserving the distribution of each predictor to assess its influence on the model performance. The third approach, drop-column importance, excludes each feature one at a time and examines how it impacts the predictive accuracy.

The characterization of the extremes in hydrology and ecosystem variables is captured based on the outputs from the model integrations assimilating remotely sensed LAI. Deficits in precipitation and root zone soil moisture from the model integrations are assumed to represent the meteorological and agricultural droughts^48,81^. Specifically, the 12-month standard anomalies in precipitation and 6-month standardized anomalies in the top 1m root zone soil moisture across 2003–2020 are used as analogs of drought. This 12 month precipitation lag window is chosen based on the convention used at the NOAA National Centers for Environmental Information to characterize long-term and persistent droughts. A six-month and two-month window is chosen for capturing agricultural droughts based on the assumed temporal limits soil moisture and vegetation memory^82–84^, respectively.

### Ancillary datasets

A number of ancillary datasets are used in the analysis and evaluation of the results presented in the article. A map representing the variation in the potential density of cattle head per ranch (certified in the *Cadastro Ambiental Rural* in the Brazilian Pantanal)^37^ is used as an index of the level of pasture in this area. This map is estimated by the Mapbiomas project^85^ (https://mapbiomas.org/), which is a multi-institutional initiative to map the land use dynamics in Brazil and other tropical countries based on information from remote sensing and local information. There are large ranches in this region with 62.3% or more than 5000 ha^37^. As shown in Fig. 1, we define four categories of pasture with cattle heads per hectare ranging from 0 to 0.03, 0.03 to 0.1, 0.1 to 0.4, and > 0.4 for very low, low, medium, and high pasture density areas, respectively.

The impact of assimilating LAI data on various water and carbon fluxes and states is evaluated by comparing the model simulations with a number of remote sensing-based global reference data products (Extended Analysis 1). Soil moisture states from the model are evaluated by comparing against the Level 2 retrievals from NASA’s Soil Moisture Active Passive (SMAP^86^) mission. In addition to soil moisture, SMAP also provides estimates of vegetation optical depth (VOD), an analog of the above ground canopy biomass^87^, derived as part of the radiometric soil moisture retrieval. Here we use both VOD and soil moisture from the SMAP SPL2SMP_E product, which is available at 9-km spatial resolution through Backus-Gilbert interpolation applied to oversampled antenna measurements^88^. To evaluate the evapotranspiration (ET) estimates, MODIS thermal infrared (TIR) data based ET from the Atmosphere-Land Exchange Inverse (ALEXI^89,90^) model is used. The 5-km resolution gridded ALEXI ET estimates are used in this study. The subsurface moisture simulations are evaluated by comparing the monthly terrestrial water storage anomalies against those from NASA’s Gravity Recovery and Climate Experiment (GRACE) and GRACE-Follow On (GRACE-FO) satellites. Specifically, we use the Release 06 GRACE^91^ Level-2 Mass Concentration blocks (mascons) data products available from the Center for Space Research (CSR) at University of Texas. This product is available at 0.25 deg spatial resolution at monthly intervals. The impact of LAI DA on carbon fluxes is evaluated by comparing the simulated Gross Primary Production (GPP), which represents the total carbon fixation by plants through photosynthesis, against two remote sensing-based estimates: (1) remote sensing retrievals of Solar Induced Fluorescence (SIF) from the Global Ozone Monitoring Experiment-2 (GOME-2) aboard the MetOp-A satellite^92,93^ and (2) MODIS reflectance-based FLUXSAT product^94^. SIF represents the amount of solar radiation absorbed by chlorophyll and reemitted as fluorescence and is considered as a functional analog of GPP. FLUXSAT is developed through a data driven modeling approach by calibrating the MODIS reflectance information to ground measurements from the FLUXNET network. For uniformity of comparison, all reference data products are interpolated to the 1-km modeling grid in these evaluations.

## Supplementary Information

Supplementary Information.
