# Supplementary material for: Changes in land use enhance the sensitivity of tropical ecosystems to fire-climate extremes
Source: Sci Rep. 2022 Jan 19;12:964. doi: 10.1038/s41598-022-05130-0 (PMC8770517; doi:10.1038/s41598-022-05130-0)
Supplement: Supplementary file 1 — Supplementary Information. [file 41598_2022_5130_MOESM1_ESM.pdf]

Supplementary Information

Analysis methods

Though all the data products used to evaluate the DA integrations are based on remote-sensing observations, their development does include modeling assumptions. Therefore, they also have associated biases of their own and cannot be considered as true validation products. Therefore, we primarily focus on two evaluation metrics that quantify the level of association between the model simulations and the reference data products: (1) Pearson correlation coefficient (R) and (2) mutual information (I). R measures the strength of the linear relationship between two variables X and Y, computed as :

$$R(X,Y) = \frac{cov(X,Y)}{\sigma_x \sigma_y}$$

Where $cov\ (X, Y)$ represents the covariance between X and Y and σ_x and σ_y represent their standard deviations. *I*, on the other hand, provides a measure of the mutual dependence between X and Y, by computing how the joint distribution of X and Y is different from the marginal distributions of X and Y. I is computed as:

$$I(X,Y) = \sum_{x \in X} \sum_{y \in Y} p(x,y) log(\frac{p(x,y)}{p(x)p(y)})$$

Where p(x,y) is the joint probability distribution of X and Y and p(x) and p(y) are the marginal probability distributions of X, and Y, respectively.

The Spearman correlation coefficient, computed as the Pearson correlation coefficient between the rank variables is used to quantify the level of association between various climate and environmental factors that affect fire occurrences. As Spearman correlation is non-parametric and does not rely on assumptions of normality, we consider it more appropriate for examining the correlation measures involving the fire occurrences. The statistical significance of the differences in correlation measures shown in Figure S3 and S5 are estimated using the Fisher Z transform[95].

The monotonic trends (increasing, decreasing, or no trend) and their statistical significance in the fraction of burn area (shown in Fig. 2) is computed using the non-parametric Mann Kendall Test[96,97], using the following statistic.

$$S = \sum_{i=1}^{n-1} \sum_{j=k+1}^{n} sign(x_j - x_i)$$

Where *x* is the time series variables and the subscripts *j* and *k* are the observation times. $sign(x_j - x_i)$ is equal to +1, 0, or -1, indicating increasing, no, and decreasing trends, respectively. In this study, the statistical significance of the trends is examined at a 10% significance level.

Extended analysis 1: Evaluation of data assimilation integrations

Figures S7 and S8 represent the changes in *R* and *I* as a result of data assimilation on various water and carbon variables. The change maps are computed as *R/I* of DA minus *R/I* of the OL, so that positive values (and warm colors) indicate improvements and negative values (and cool colors) indicate degradations from DA. Both Figures S7 and S8 indicate the beneficial impacts of LAI assimilation. There are significant improvements in the modeled LAI when compared to SMAP VOD data with a domain averaged increase in *R* and *I* of 0.28 and 0.3, respectively. This confirms that the assimilation of LAI enables better characterization of vegetation variations in this region. These improvements in modeled LAI lead to small, but beneficial changes in soil moisture and ET. Compared to SMAP soil moisture, there is a domain averaged improvement of 2.5% and 2.3% in R and *I*, respectively. LAI DA leads to a domain average 6.6% (5.2%) improvement in the *R(I)* of ET against ALEXI. Consistent with the spatial patterns in Figures S7 and S8, 4.1% (3.7%) improvement in domain averaged TWS anomalies are obtained as a result of LAI assimilation. Compared to the improvements in soil moisture, ET, and TWS, a larger level of improvements is observed in the GPP evaluations. In the comparisons to FLUXSAT, a domain averaged 21% improvement in the correlation of simulated GPP is obtained with DA. Similarly, a domain averaged improvement of 28% is obtained in the FLUXSAT comparisons with mutual information. Finally, the GOME-2 SIF comparisons also show domain averaged improvement of 11.8% and 6.8% in GPP with *R* and *I* metrics, respectively. These evaluations confirm that the improved representation of vegetation conditions from LAI DA leads to systematic improvements in the simulated water and carbon states. These results are consistent with the findings from similar prior studies over other regions of the world[75,76,98]. We employ the DA integration for all the main analysis as it consistently performs better than the OL.

Data availability

The underlying data used to generate the Figures presented in the manuscript can be accessed from http://doi.org/10.5281/zenodo.5090119.

Code availability

The NASA Land Information System (LIS; version 7.4.5) software used for the modeling, data assimilation and analysis of the results is freely available from https://github.com/NASA-LIS/LISF

Supporting Figures

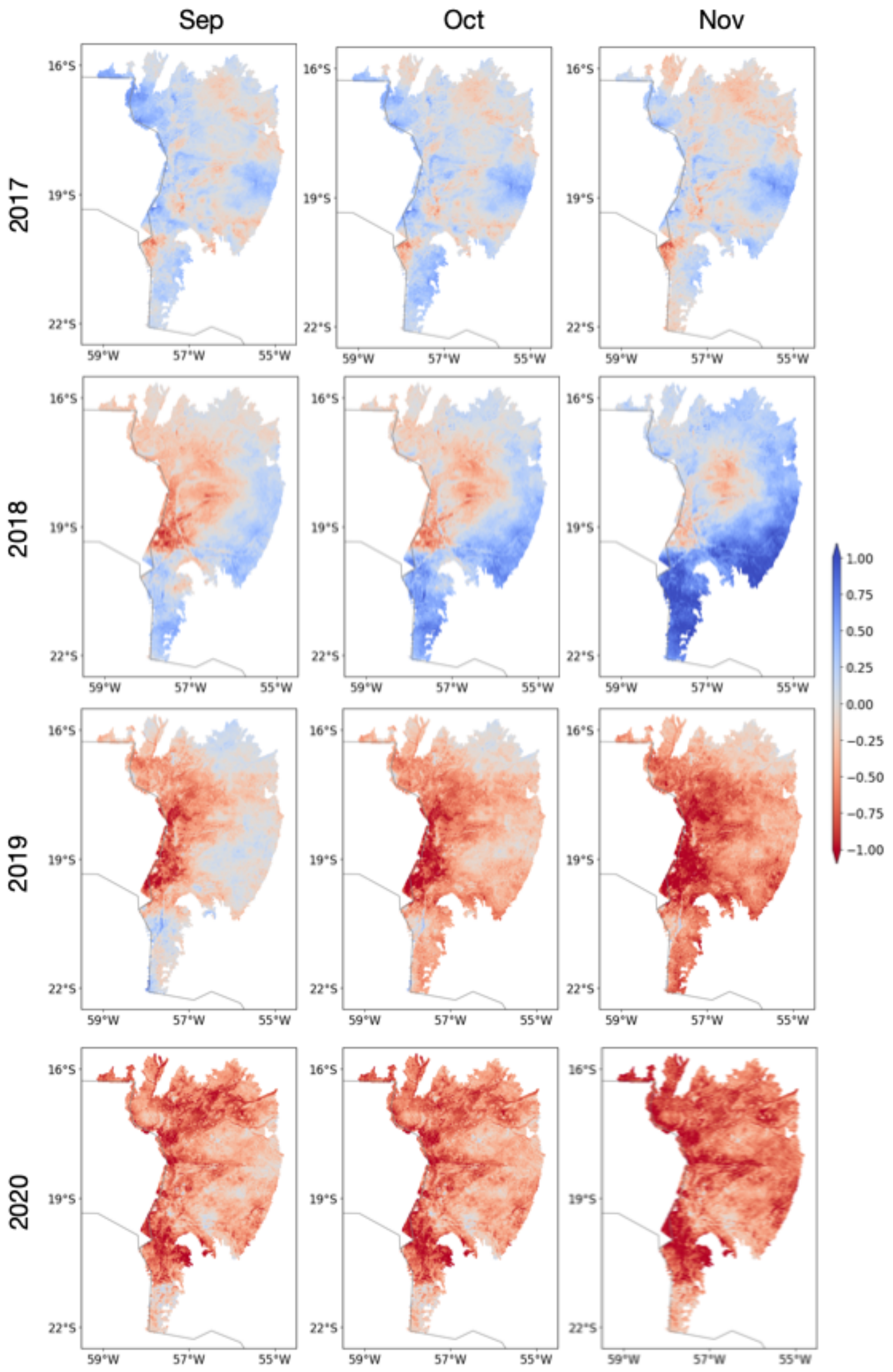

Supplementary Figure S1: Spatial maps of standardized 6-month RZSM anomalies during Sep-Nov for years 2018 to 2020.

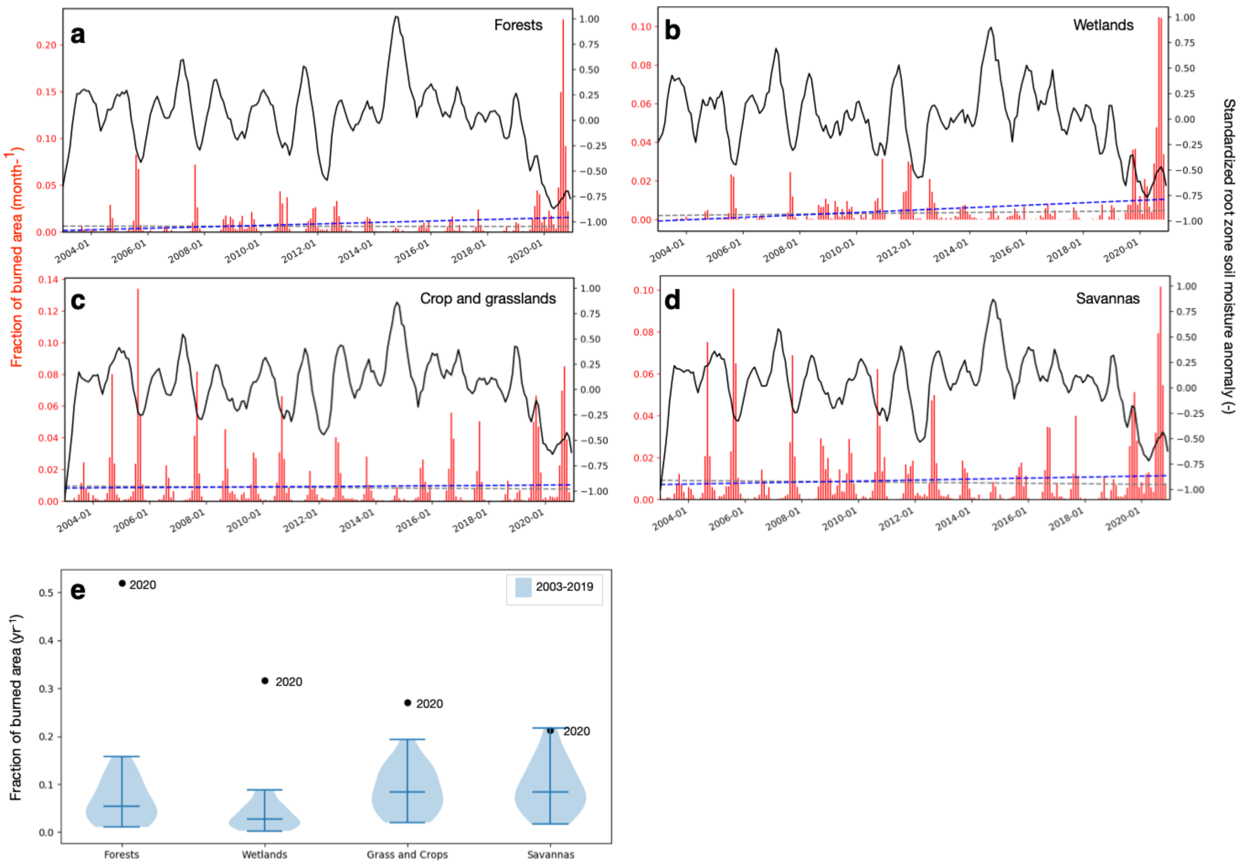

Supplementary Figure S2:Time series of the standardized anomalies of antecedent 6-month root zone soil moisture (black lines) and the fraction of burned area (red bars) over four major land cover types (Panels a-d). The dashed blue and gray lines show the linear trend of the fraction of burned area over the 2003-2020 and 2003-2019 time periods, respectively. Panel e shows the distribution of annual fraction of burned area across 2003-2019 and from 2020.

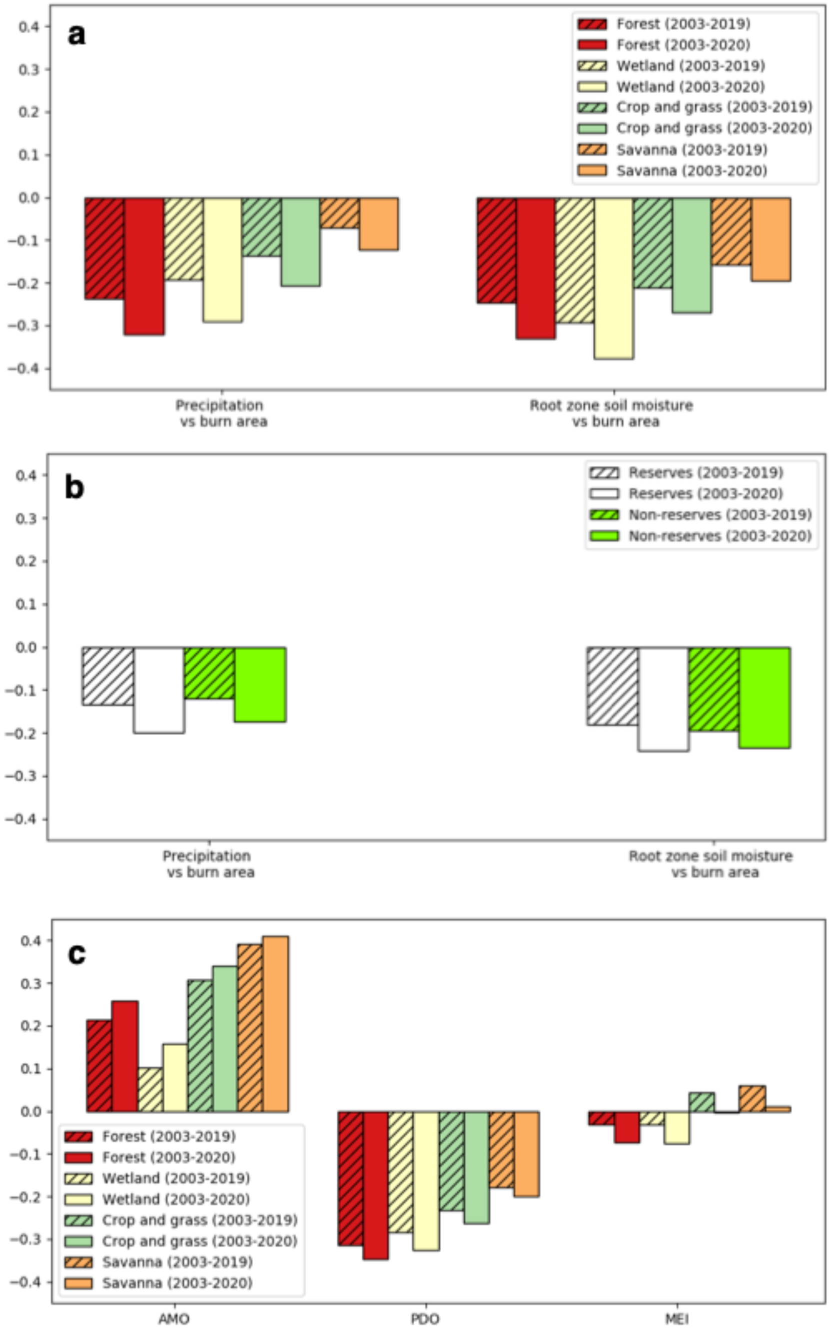

Supplementary Figure S3: (a) shows the Spearman correlation between standardized anomalies in the 12-month antecedent precipitation and 6-month root zone soil moisture against the percent of burn area, stratified over the four major land cover types for two different time periods (2002-2019, and 2002-2020; (b) is similar to a) except for reserved and non-reserved areas,); (c) shows the Spearman correlation between the climate indices of AMO, PDO, and MEI against the percent of burn area for 2002-2019 and 2002-2020 time periods.

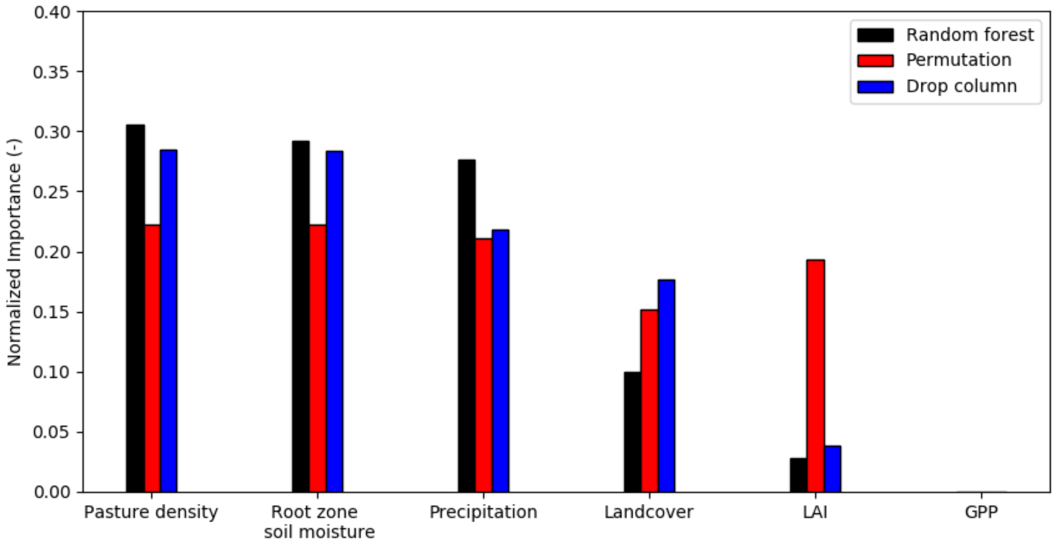

Supplementary Figure S4: Normalized feature importance estimates from the random forest model, permutation feature importance, and drop column feature importance approaches.

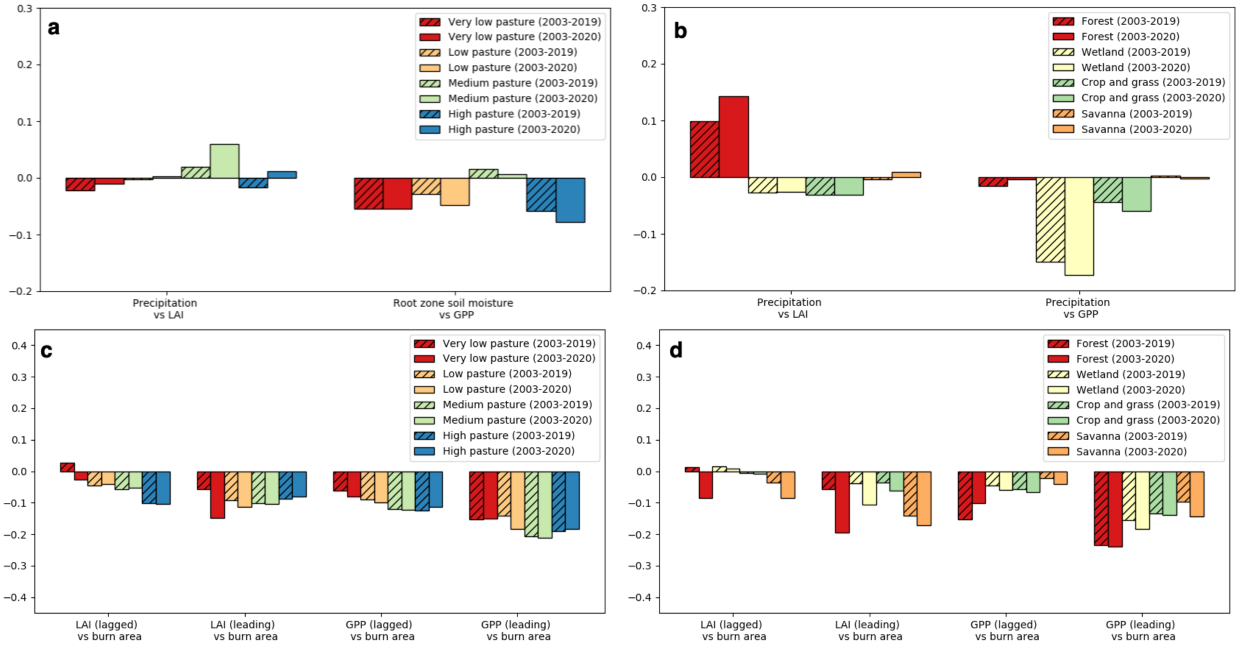

Supplementary Figure S5 : Panels a and b show the Spearman correlation between the antecedent 12-month precipitation and 2-month LAI/GPP, stratified over areas with different pasture density and land cover type, respectively. Panel c shows the Spearman correlation between LAI and GPP against the fraction of burn area for the 2002-2019 and 2002-2020 time periods, computed separately for the lagging (using antecedent 2-month anomalies) and leading (2-month anomalies in the following months), over areas with different levels of pasture density. Panel d is similar to Panel c, computed over the four major land cover types.

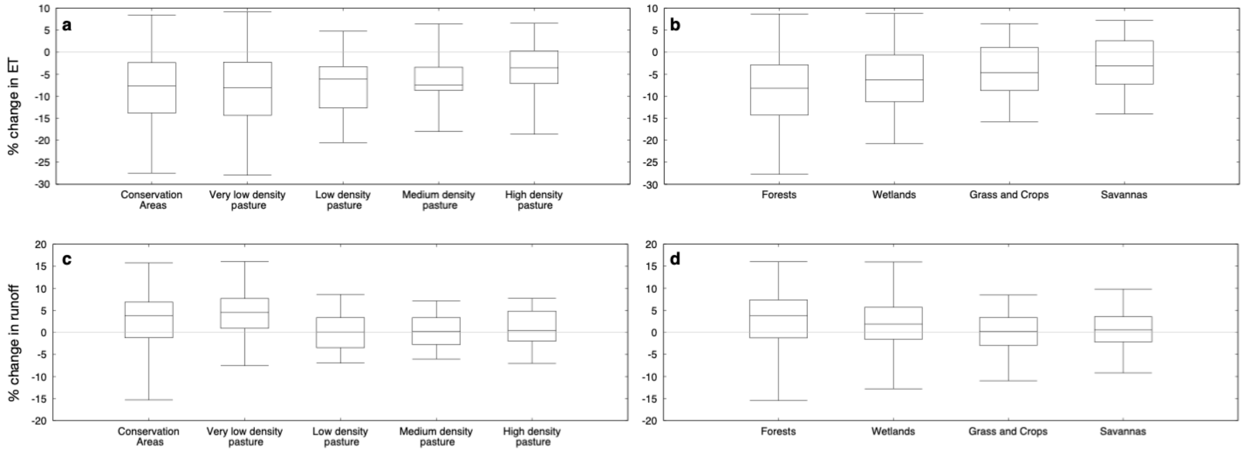

Supplementary Figure S6: Panels a and b show the distribution of changes in ET during the post-fire time period of 2020 relative to the scenario with climatological vegetation conditions, stratified over areas with different land management and land cover biomes. Panels c and d show similar impacts in runoff.

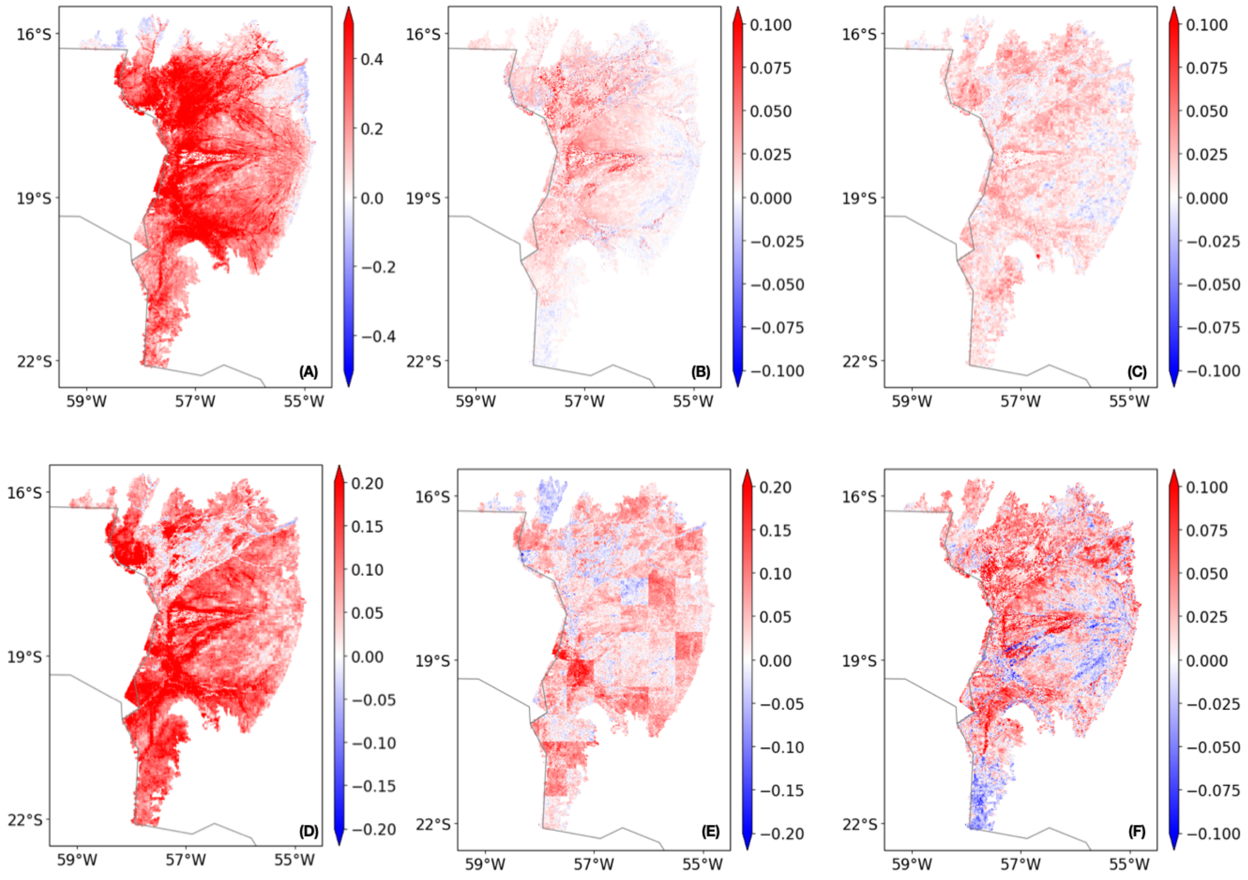

Supplementary Figure S7: Changes in Pearson correlation R (expressed as R(DA) minus R(OL)) in (A) LAI compared to SMAP VOD, (B) soil moisture compared to SMAP soil moisture, (C) ET compared to ALEXI ET, (D) GPP compared to FLUXSAT GPP, (E) GPP compared to GOME-2 SIF, and (F) TWS compared to GRACE-TWS.

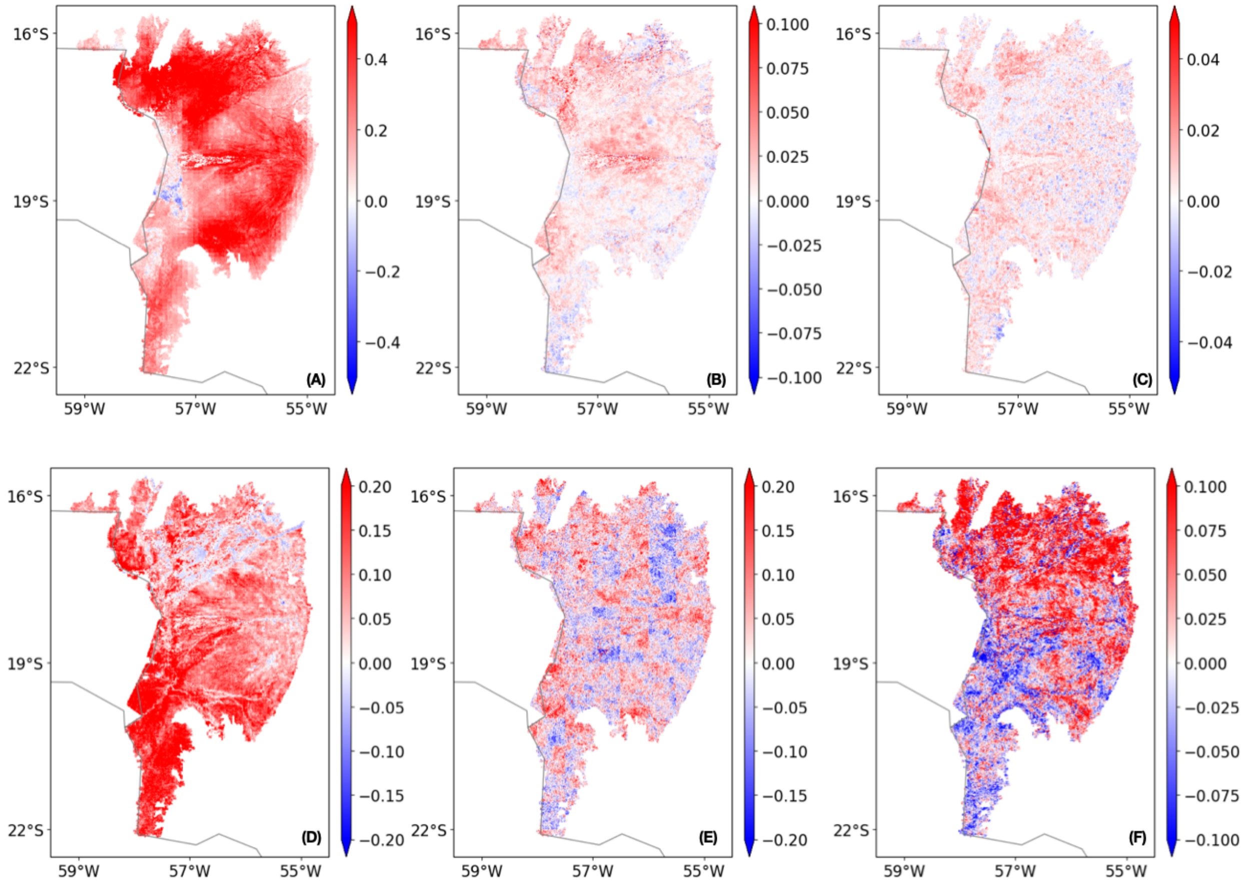

Supplementary Figure S8: Changes in I (expressed as I(DA) minus I(OL)) in (A) LAI compared to SMAP VOD, (B) soil moisture compared to SMAP soil moisture, (C) ET compared to ALEXI ET, (D) GPP compared to FLUXSAT GPP, (E) GPP compared to GOME-2 SIF, and (F) TWS compared to GRACE-TWS.
